# A Smartphone Lightweight Method for Human Activity Recognition Based on Information Theory

**DOI:** 10.3390/s20071856

**Published:** 2020-03-27

**Authors:** Hendrio Bragança, Juan G. Colonna, Wesllen Sousa Lima, Eduardo Souto

**Affiliations:** Instituto de Computação, Universidade Federal do Amazonas, Manaus CEP 69067-005, Brazil; juancolonna@icomp.ufam.edu.br (J.G.C.); wesllen@icomp.ufam.edu.br (W.S.L.); esouto@icomp.ufam.edu.br (E.S.)

**Keywords:** human activity recognition, symbolic representation, discrete domain, time series classification, information theory, inertial sensors

## Abstract

Smartphones have emerged as a revolutionary technology for monitoring everyday life, and they have played an important role in Human Activity Recognition (HAR) due to its ubiquity. The sensors embedded in these devices allows recognizing human behaviors using machine learning techniques. However, not all solutions are feasible for implementation in smartphones, mainly because of its high computational cost. In this context, the proposed method, called HAR-SR, introduces information theory quantifiers as new features extracted from sensors data to create simple activity classification models, increasing in this way the efficiency in terms of computational cost. Three public databases (SHOAIB, UCI, WISDM) are used in the evaluation process. The results have shown that HAR-SR can classify activities with 93% accuracy when using a leave-one-subject-out cross-validation procedure (LOSO).

## 1. Introduction

Recent advances in sensing technologies have made smartphones one of the most promising devices to the real-time monitoring of human behaviors in different domains such as health care, fitness track, behavior detection, elderly care, and rehabilitation [1,2,3,4]. In the health area, for instance, the recognition of physical activities from smartphone sensor data have contributed to avoiding negative outcomes linked with a sedentary lifestyle. For example, monitoring of time a person spends sitting can be a useful parameter for treating diseases such as obesity, diabetes, cancer, and cardiovascular disease [3].

In general, the task of human activity recognition (HAR) consists mainly of four steps: sensing (data acquisition), segmentation, feature extraction, and classification [2,5,6]. While all these steps are important, feature extraction and selection are the core of most studies in the HAR area [1,6,7]. The feature extraction phase aims to find a high-level data representation from sensors signals which are used by machine learning methods to generate activity recognition models [8].

Early researches have focused on shallow machine learning methods that use a hand-crafted process to obtain significant features from sensors data. In this process, the data gathered from sensors should be transformed by a specialist into a new domain that represents the data into a high-level representation [2,5,9,10]. A disadvantage of these shallow approaches is that feature extraction depends on the expertise of an expert and therefore is a lengthy process [11].

The need for more robust techniques for learning features automatically from sensor data and minimizing the participation of the specialist is a trending topic in HAR. In this context, Deep Neural Networks (DNNs) have been used [11,12,13,14,15,16]; however, although this approach is promising, DNNs present high computational cost when compared with traditional approaches, which makes them unfit for real-time applications implemented on mobile devices with limited processing, memory, and battery resources [4,17].

Alternatively, symbolic representation algorithms are used to overcome problems mainly related with high computation cost in time series domain [18]. The symbolic algorithms transform raw sensor data into useful and compact information to facilitate the learning process of machine learning algorithms. The advantages of discrete domain features include the ability to reduce the data dimensionality and data numerosity naturally during the feature extraction, allowing a massive amount of data to be reduced to a reasonable and representative number of symbols. This contributes to reducing the complexity and computational cost of HAR solutions, opening doors for the use of new HAR strategies in the context of smartphones with limited memory and processing resources.

Examples of these algorithms are Symbolic Aggregate Approximation (SAX) proposed by Lin et al. [19,20], Symbolic Aggregate Approximation in Vector Space Model (SAX-VSM) proposed by Senin and Malinchik [21], Symbolic Fourier Approximation (SFA) proposed by Schäfer and Högqvist [22], Bag-of-SFA-symbols (BOSS) proposed by Schäfer [23] and Multivariate Bag-Of-SFA-Symbols (MBOSS) proposed by Montero Quispe et al. [24].

In this article, we propose a low-cost solution called Human Activity Recognition based on Symbolic Representation HAR-SR), which is capable of extracting features from sensors data using symbolic representation algorithms by transforming a time series, represented by real values obtained from sensors, into a set of symbols belonging to the discrete domain. Unlike the approaches that use the Term Frequency – Inverse Document Frequency (tf-idf) model [25], e.g., SAX-SVM model, BOSS model and MBOSS model, which generally have a larger feature set, the advantage of our method is that the feature set is significantly smaller. The data representation in the discrete domain [19,23] is combined with information theory techniques [26,27] which arrange the input signals in a much smaller space than the original one. This generates a low-cost classification model in terms of computational performance.

The objective of this article is to improve Human Activity Recognition (HAR) solutions based on smartphones instrumented with inertial sensors with the use of scalable and low-cost strategies. The main contributions of this article are: (a) a low-cost feature learning approach to transform time-series into a symbolic domain; (b) new features introduced to the HAR domain based on complexity measures from information theory that characterizes properties of a time series; (c) a feature set significantly smaller than those generated by the similar models found in the literature [21,23,24]. The results show that HAR-SR performs similarly to the state-of-the-art. However, it presents a low computational cost when compared to other traditional models [9,10].

The remainder of this paper is organized as follows: Section 2 presents the background for Human Activity Recognition. Section 3 details the concepts related to the symbolic representation of sensor signals. In this section, we discuss the discretization algorithms and the process of feature extraction of the discrete domain. Section 4 presents the proposed method. Section 5 and Section 6 presents the experimental protocol and the results. Section 7 presents the work related to this research. Finally, Section 8 presents the conclusions of this work.

## 2. The Human Activity Recognition Process Overview

**Figure 1:**
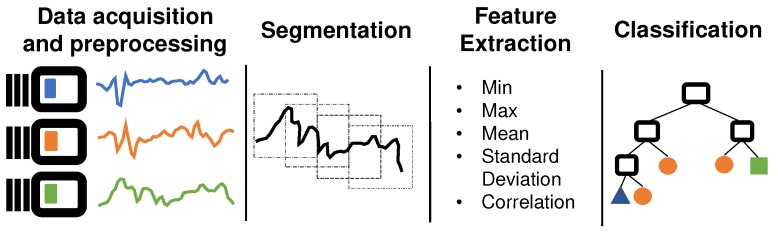
The common methodology used in HAR: data acquisition, segmentation, feature extraction, and classification.

The smartphones are devices capable of real-time monitoring of human behaviors through several embedded sensors such as accelerometer, gyroscope, microphone, camera, and GPS [1]. In general, the methodology used to find patterns in the sensors signals and associate them to human activities is divided into four main stages [2,5,6,24], as shown in Figure 1.

In the data acquisition stage, the motion sensors are often used (e.g., accelerometer and gyroscope) because they give information about the angle, vibration, rotation, and oscillation of a smartphone. These data can be considered a direct reflection of a user’s actions and/or of the physical environment in which the device is located [28,29,30]. The data are collected continuously and should be divided into smaller segments in a process called segmentation. The segment size influences the amount of data needed to represent the activities.

The windowing approaches are normally used for segmentation. In the simplest segmentation technique, the signals are split into windows of a fixed size, and with no overlap in the between-segments data collected; however, the data associated with a particular activity can be split between different segments, which can result in the loss of important information. To overcome this problem, the sliding window segmentation techniques are used to add some overlap in the process of segmentation.

Subsequently, each segment is transformed into a high-level representation of sensor signals in a process called feature extraction. This process is essential because the classifiers do not work very well if they are applied directly to the sensor signal data [8,31,32]. There are two ways of extracting features from sensors signals data: hand-crafted feature extraction and automatic feature extraction (feature learning). In the first approach, the features are manually engineered by the data scientist, while in the second approach, the features are automatically obtained from a machine learning algorithm, using deep neural networks, for example.

In general, the shallow networks are modeled by features extracted from time, frequency, or discrete domain. These features are simple to understand and have low computational complexity, very suitable for mobile devices [7,13]. The disadvantage of hand-crafted feature extraction is that resources created or selected manually are time consuming, domain-specific, and they require specialized knowledge. On the other hand, these features can be generated automatically by using deep structured models. Most feature learning approaches used in the feature extraction phase are based on autoencoders and Convolutional Neural Networks (CNNs) [4,15,17,33].

The features extracted are used by machine learning algorithms to generate an activity recognition classification model [8]. Different machine learning algorithms can be used to create the recognition models such as decision tree, naive Bayes, support vector machine (SVM), artificial neural networks (ANNs), logistic regression, KNN (K-Nearest Neighbors) and Deep Neural Networks (DNNs) [8,34].

The DNNs are a more recent class of machine learning algorithms used in HAR systems. As a feature learning method, the DNNs eliminate or minimize the need for a dedicated feature extraction phase because the entire process of feature extraction and classification is performed within the network hidden layers. The DNNs have a high computational cost when compared with traditional approaches, which makes them unfit for real-time applications implemented on mobile devices with low computational power (CPU and low-performance GPU) [35].

An alternative to deal with the problems of high computational cost derived by the use of deep structured models is to adopt a discrete representation of features. Symbolic representation algorithms have been used to reduce data dimensionality and noise of the time series to improve the classification stage of the machine learning algorithms [18,19,20,22,36]. These algorithms are also known as histograms-based or codebook based, which use frequency counts of recurrent patterns and then build classifiers based on the resulting histograms [18,21,24,37,38]. This is a recent approach that has shown good results in problems involving time series classification.

## 3. The Discrete Domain of Sensors Data

The discrete domain algorithms can transform the sensor signal, represented by a time-series, into a set of symbolic patterns. The symbolic patterns can be interpreted as frequency distribution, a new high-level representation of the signals. This new form of representation has some advantages that are intrinsic to its transformation processes, such as dimensionality reduction and noise reduction. In literature, the discrete domain algorithms more used are the Symbolic Aggregate Approximation (SAX) [19] and Symbolic Fourier Approximation (SFA) [22]. These algorithms are divided into two phases:

These algorithms are divided into two phases that are represented by two parameters:

(a) Approximation: it is a mapping of a time series in a low dimension space, represented by real values. This parameter consists on word size, ω∈N, which represents the number of values for the approximation. The smaller the word size, the greater the reduction of noise, but the loss of information increases;
(b) Quantization: Each real value obtained in the approximation process is mapped to a discrete value, which is interpreted as a symbol. This parameter is defined as the alphabet size α∈N, which is used in quantization. A small alphabet also results in a strong noise reduction.

The approximation and quantization can result in different types of representations for the same time series, depending on the algorithm adopted. These processes will be described with more details in the next sections.

### 3.1. Symbolic Aggregation Approximation

The Symbolic Aggregation Approximation (SAX) is a discretization algorithm that transforms an arbitrary time series *T* of size *N*, represented by real values, into a set of arbitrary symbols of size *M* in a discrete domain [19].

In the approximation phase, the SAX applies the standard normalization on time series and use the numerical approximation algorithm Piecewise Aggregate Approximation (PAA) for dimensionality reduction. A time-series *T* of size *N* is divided into *M* subsequences of equal size, where each subsequence is represented by the mean of values. Since transformed a time series into the PAA representation, we apply a further transformation to obtain a discrete representation.

**Figure 2:**
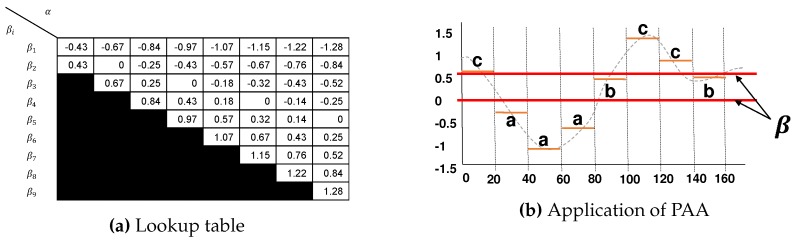
(**a**) The lookup table shows the breakpoints that divide the Gaussian distribution into an arbitrary number of equiprobable regions. (**b**) The application of the PAA in a time series (N = 160) resulting in a new signal (M = 8). The new signal is composed of all values *t_i_*, which are computed at each interval.

Finally, in the quantization phase, each of PAA coefficients is mapped into a letter ω of an alphabet α using a lookup table (Figure 2a) which defines a set of breakpoints that divide the normalized time series values distribution space into α equal-sized regions, assuming the Gaussian distribution [19,39]. Figure 2b shows the statistical table used in the definition of breakpoints.

The SAX word generation process is considered a simple and computationally inexpensive process when compared to more robust algorithms, such as SFA, presented in the next section.

### 3.2. Symbolic Fourier Approximation

The Symbolic Fourier Approximation (SFA) is one of the most sophisticated discretization algorithms for time-series found in the literature [18,22].

**Figure 3:**
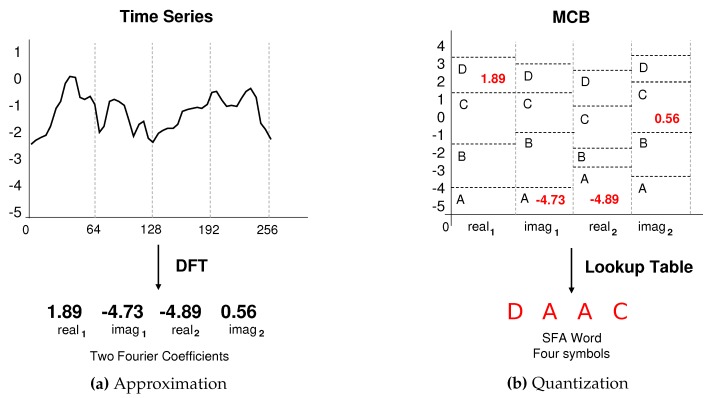
A time series *T* is approximated using the Discrete Fourier Transform (DFT) coefficients. In the quantization phase, the SFA uses the Multiple Coefficient Binning (MCB) to define the best breakpoints and finally generate the word ‘DAAC’ of size 4 and alphabet of size 6 [22].

In the approximation phase, the SFA algorithm uses the first coefficients of the Discrete Fourier Transform (DFT). The number of Fourier coefficients is associated with the SFA word parameter, l∈N. Dynamically adding or removing Fourier coefficients adapts the degree of approximation of the algorithm. The first coefficients are the ones that capture the most important frequency components. For this reason, adding more coefficients will not affect the approximation accuracy significantly. In addition, the coefficients are calculated only once, and there is no need to recalculate them unless it is necessary to change the approximation level. Figure 3a shows the original subsequence transformation into the real and imaginary coefficients extracted from DFT [22].

In the quantization phase, the SFA algorithm uses a technique called Multiple Coefficient Binning (MCB) to generate a lookup table, which is based on a set of time series samples. The MCB define a set of regions, where each DFT coefficient represents a breakpoint. Each region of the breakpoint, called bin, has different intervals for each column of coefficients. These differences make the SFA better adapt to the data variation and also reduce the risks of information loss [22]. Figure 3b shows the quantization phase which uses the MCB table to define the best breakpoints and generate the symbolic representation of a time series.

After the training, the MCB can be used in the process of transformation of a time series in a symbolic representation. In practical terms, the SFA algorithm works similarly to SAX, but in the approximation phase, it uses the Fourier transform instead of the PAA algorithm. Besides, the SFA breakpoint table is dynamic, which fits the data based on the training set, instead of SAX that uses a fixed and predefined table. The mentioned changes generate an additional cost to the method and an additional step to generate the lookup table. However, this additional cost is offset by better results compared to the SAX in several scenarios [18,23].

### 3.3. Time-Series Bag-of-Patterns Representation

**Figure 4:**
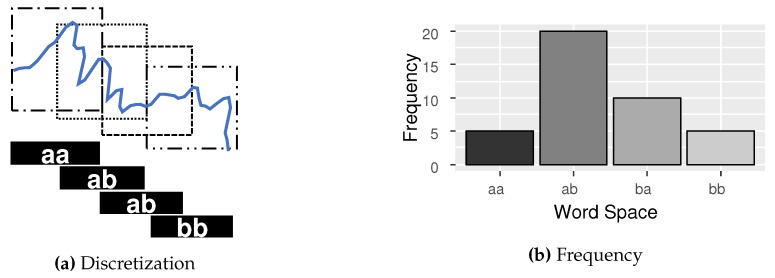
The discretization process of a time series to obtain a frequency distribution. The time series is segmented through the sliding window technique, and then discretized by some symbolic representation algorithm such as SAX or SFA.

For finding the most representative patterns in time series, it is common to use a methodology called Bag-of-Patterns (BOP) [20,37]. The BOP combines the symbolic representation algorithms with the sliding window techniques. This allows the symbolic algorithms, such as SAX, to extract substructures and more representative patterns from the same time-series. The result of this combination becomes a set of symbols (frequency distribution) and not a single representation, as shown in Figure 4.

As shown in Figure 4a, the algorithm begins by extracting the sliding windows of size w from the time series *T*. Then, each sliding window is normalized by z-score. Finally, the symbolic transformation is applied in each of the values obtained in the sliding window process. A histogram is constructed based on the frequency of symbols. The histogram $P:\alpha^{\omega }\to N$ is a function of the space of symbols $\alpha^{\omega }$ for natural numbers. The numbers represent the occurrences of a symbol. This unordered symbol histogram is known in other domains as bag-of-words (BOW).

A Probability Distribution Function (PDF) is commonly used to quantify a frequency distribution that represents the time series. The distribution can be treated as a set of features by the classification methods. From the same distribution, it is also possible to apply other techniques to obtain new characteristics of higher quality.

For example, the vector space model called Term Frequency – Inverse Document Frequency (tf-idf) [25] can be used to obtain the vector of weighted frequencies (or weights) from a histogram. In this model, the words that are syntactically similar and the furthest words are syntactically distant. In the model tf-idf, each of the symbols receives a weight according to its importance in the syntactic context. This model succeeds in representing the syntactic relationship between symbols and their importance since the weight applied to each symbol generates a vector of characteristics better than that presented only by the frequency of symbols [40].

Adaptations of this model applied in the discrete domain that applies tf-idf in the frequency distribution are presented in Senin and Malinchik [21] with SAX-SVM model; Schäfer [37] with the Bag-of-SFA-symbols (BOSS) model; Montero Quispe et al. [24] with Multivariate Bag-Of-SFA-Symbols (MBOSS) model. Each one of these methods uses different techniques that result in different representations.

This work proposes another solution that introduces information theory quantifiers as a new feature extracted from a frequency distribution. These quantifiers of information theory can be defined as measures that characterize properties of a distribution, allowing the extraction of information from time series, where determinism and stochastic are two extremes of the process [27]. The statistical complexity measure of a probability distribution can be used as a feature by machine learning algorithms. Unlike the approaches that uses tf-idf models, e.g., SAX-SVM model, BOSS model and MBOSS model, the advantage of our method is that the feature set is significantly smaller.

## 4. HAR-SR: Human Activity Recognition Based on Symbolic Representation

The HAR-SR (Human Activity Recognition based on Symbolic Representation) was designed to classify multivariate time series. The multivariate time series are collections of univariate time series that arise when multiple interconnected data sources captured over time. The HAR-SR maps the implicit features of sensor’s signals to a discrete domain as a frequency distribution. From this distribution, this research presents a new feature set for classification models, derived from information theory, such as Shannon’s Entropy, Jensen-Shannon Divergence, and the Statistical Complexity Measure [27,41]. By the end, a supervised learning algorithm is used to create a model for activity recognition.

**Figure 5:**
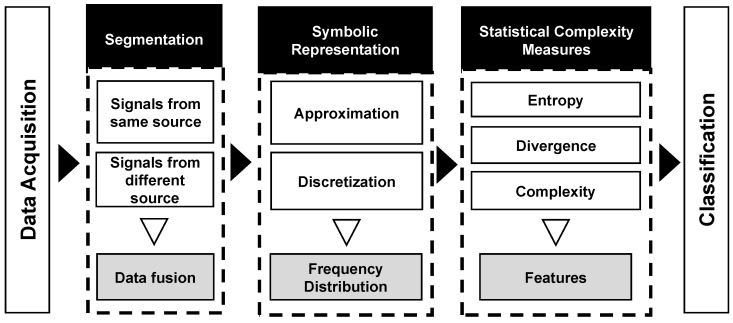
Overview of the HAR-SR method.

The HAR-SR recognition process has five main steps, as shown in Figure 5. (a) data acquisition; (b) signal segmentation phase, which makes use of data fusion techniques; (c) the symbolic representation phase, which learns patterns from time series; (d) the feature extraction stage presents new features, that are not yet explored in the HAR literature; (e) the classification phase creates a classification model using a machine learning method for activity recognition.

### 4.1. Data Acquisition, Segmentation and Data Fusion

Some discretization methods, such as SAX, do not accept multidimensional time series and therefore cannot directly receive data from motion sensors. The choice of a data fusion technique on the segmentation process of the multidimensional time series should resolve this problem. In the data fusion process, we have used different techniques, such as magnitude and the Principal Component Analysis (PCA), both aggregation methods [42]. In addition to these methods, a simple concatenation technique is used to obtain a unidimensional signal $T=(t_{i},...,t_{n})$, $t_{i}\in R$, but with the same size as the original signals. A limitation of this approach is that no dimensionality reduction is applied in this concatenation method.

### 4.2. The Symbolic Representation of Sensors Signals

We use the following steps in the discretization process of a time series: (a) using the sliding window technique for extracting substructures, or segments, from a time series. Given a time series *T*, a segment *S* is a time series with *w* contiguous values starting at offset *a* in *T*, where $S\left(a,w\right)=\left(t_{a},\ldots ,t_{a+w-1}\right)$, with 1≤a≤n−w+1. In this process, n−w+1 sliding windows of length *w* can be extracted from a time series *T* of length *n*; (b) each segment of the time series goes through a process of symbolic approximation, whose objective is to reduce the dimensionality of the segment and to remove noises. (c) then, a search table is used in the discretization process to obtain the symbolic representation of the segment; (d) finally, each discretized segment is used to obtain a frequency histogram of the symbols.

The result of the discretization process is a frequency distribution for each time series from the dataset. The histogram feature space is formed by all combinations of letters that generate a word. It can be visualized as a matrix where rows are related to the words space and the columns to the segments. If a specific word is already in a histogram, then the frequency value is nonzero. Otherwise, the value is equal to zero. Each histogram has an activity label that will be used for classification purposes.

When all histograms are computed, they can be used directly by a classification model as a feature set. In this work, we employ a new feature set based on metrics extracted from information theory as statistical quantifiers such as entropy, divergence, and complexity for the classification task.

### 4.3. A New Feature Set from Information Theory

The HAR-SR presents a new feature set for classification algorithms using information theory concepts. In the HAR domain, the Statistical Complexity Measure (SCM) is expressed in the amount of information stored by the time series. Through this measure, it is possible to define quantifiers associated with the activities performed. If the time series is transformed into a symbolic histogram, then the complexity of the time series can be accessed by the information contained in any symbol. This information is used to quantify the degree of order and disorder of the time series.

There are different measures of complexity to compare and distinguish behaviors in time series, such as periodic, chaotic, and random behaviors, as shown [41,43]. These measures should be selected according to characteristics of the data, such as stationarity, size of the time series, parameters variation, and noise level. In all the cases, the global aspects of the time series are captured in some way, but the different methods are not equivalent in discerning all the relevant physical details [44]. The SCM adopted in this work uses the measures suggested in Rosso et al. [44], which combines the concept of disorder, obtained with the Shannon entropy, and the concept of imbalance, obtained by the divergence of Jensen-Shannon.

The Shannon entropy and the divergence of Jensen-Shannon characterize properties of a probability distribution, which can be obtained using the BOP-SAX model, for example. Choosing a proper method for obtaining probability distribution from time series is not trivial since each method uses different techniques that result in different representations that must be chosen according to problem domain [27,44].

The Shannon’s entropy [45] is commonly adopted in dealing with information content. It measures the information contained in a distribution related to a time series. To compute the entropy, the histogram P must be normalized, as shown in Equation (1), where $p_{i}$ is the frequency value of the ith symbol of the word space Ω of size η.

(1) $$P=\frac{p_{i}}{\sum_{i=1}^{\eta }p_{i}}$$

**Figure 6:**
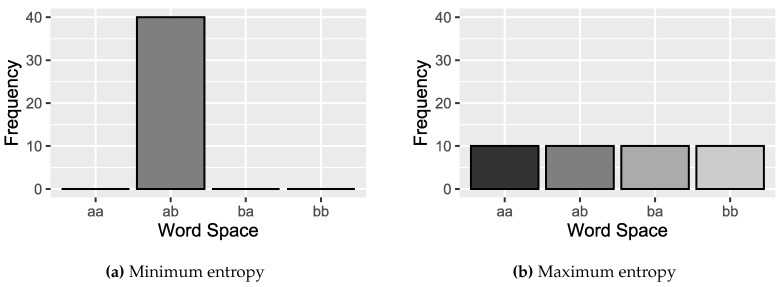
The minimum entropy can be represented by the distribution on the left, where *H*[*P*] = *H_min_* = 0. The uniform distribution *P_e_*, right in the figure, also called the uniform histogram (*H_ref_*), represents a random series in which all occurrences have the same probability value (e.g., a random noise). In the example there are four symbol possibilities, each with probability $p=\frac{1}{4}$.

Defining the probability distribution, where $P=\{p_{i};i=1,...,\eta \}$ with $\sum_{i=1}^{\eta }p_{i}$, where Ω is the word space of size η. Then, the Shannon Entropy is defined as shown in Equation (2).

(2) $$H\left[P\right]=-\sum_{i=1}^{\eta }p_{i}\ln\left(p_{i}\right),$$

where $p_{i}$ represents the relative frequencies of all possible symbols of the space Ω. When $H\left[P\right]=H_{min}=0$, the prediction that the result i will occur is of complete certainty. Figure 6a shows an example where the value H[P]=0, since the occurrence of the ‘ab’ symbol, has a maximum probability value. On the contrary, we have the least knowledge in the case of a uniform distribution $P_{e}=\left\{p_{i}=\frac{1}{\eta };i=1,\ldots ,\eta \right\}$, since each result has the same possibility to occur, and the uncertainty is maximum, $H\left[P_{e}\right]=H_{max}=\ln\eta$ (Figure 6b).

Given the two extreme situations, it is interesting to use the normalized Shannon entropy defined in Equation (3), where 0≤H≤1, and $S_{max}$ is the word space of the histogram *P*.

(3) $$H\left[P\right]=\frac{H[P]}{S_{max}}$$

**Figure 7:**
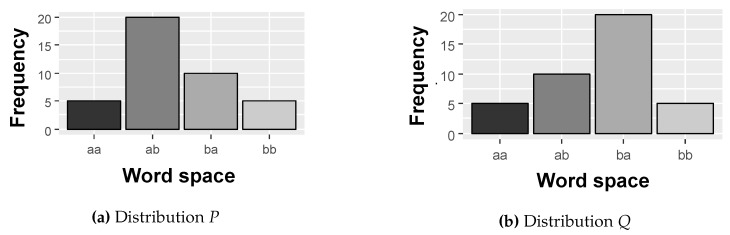
The distributions *P* and *Q* are different but have the same entropy value *H* = 0.875 (normalized entropy value).

In some cases, two different distributions *P* and *Q* may have the same entropy value, as shown in Figure 7. To solve this problem, we use the divergence calculation, which will be the second statistical measure adopted in this research to calculate the SCM. In the theory of probability and statistics, the Jensen-Shannon divergence is a measure of similarity between two probability distributions *P* and *Q* [44].

The unbalance term is used to refer to the divergence between two histograms. The first histogram *P* represents a time series and the second is the uniform histogram $P_{e}=\left\{p_{i}=\frac{1}{\eta };i=1,\ldots ,\eta \right\}$ that is the stationary state of the system. After this definition, the divergence is defined according to Equation (4).

(4) $$Q_{j}\left[P,P_{e}\right]=Q_{0}H\left[P,P_{e}\right]=Q_{0}H\left\{\begin{array}{c} \left[\frac{P+P_{e}}{2}\right]-\frac{H[P]}{2}-\frac{H[P_{e}]}{2} \end{array}\right\},$$

where $Q_{0}$ is a normalization constant such that $0\leq Q_{j}\leq 1$, which can be obtained through Equation (5). This value is obtained in a totally deterministic situation.

(5) $$Q_{0}=-2{\left\{\begin{array}{c} \frac{\eta +1}{\eta }\ln\left(\eta +1\right)-\ln\left(2\eta \right)+\ln\left(\eta \right) \end{array}\right\}}^{-1}$$

Finally, the complexity *C* combines the theory of disorder, from Shannon *H* entropy, with the concept of imbalance $Q_{j}$, from Jensen-Shannon divergence [46]. It assigns low values to both uncorrelated random data (maximum Shannon entropy) and perfectly ordered data (Shannon’s null entropy). So, if we have an ordered sequence, such as a simple oscillation or trend, the statistical complexity would be low, and so would an unordered sequence, such as uncorrelated white noise (a random signal with equal intensity at different frequencies). The characterization of the data is more difficult for series located between the two given extremes and, therefore, the complexity would be greater [27]. Thus, the statistical complexity is defined according to Equation (6).

(6) $$C\left[P\right]=Q_{j}\left[P,P_{e}\right]H\left[P\right]$$

**Figure 8:**
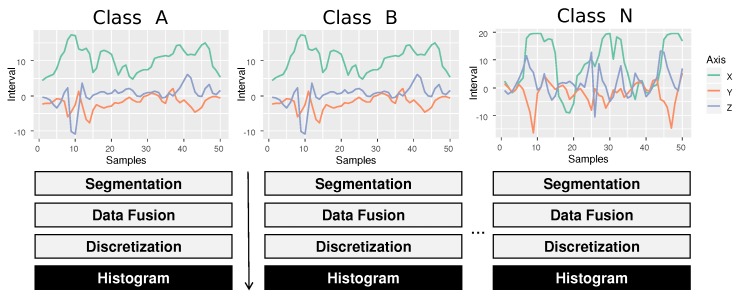
Process of generating the reference histograms for *N* classes.

For multiclass classification problems using only the three statistical measures may not be enough for good classification performance. To resolve this problem, this research also uses other interpretations for divergence measures beyond the uniform. Now the divergence value will be influenced by the number of problem classes, as shown in Figure 8. The measure of entropy remains unchanged because it depends only on the frequency distribution of a given series.

The process of generating new reference histograms for each class follows the following steps: (a) all time-series that belonging to a specific class are grouped into a set. For example, the time series of class *A* are grouped $R_{A}=\left\{T_{1}^{A},T_{2}^{A},\ldots ,T_{N}^{A}\right\}$; (b) the frequency histogram $P_{A}=\left\{P_{1}^{A},P_{2}^{A},\ldots ,P_{N}^{A}\right\}$ of each time series of the set $R_{A}$ are obtained, according to Figure 8; (c) the frequency values of each *P* symbol are summed, resulting in a single histogram: the reference histogram for class A. For example, all frequencies for the ${}^{\prime }aa^{\prime }$ symbol of the set $P_{A}$ are summed, then for the ${}^{\prime }ab^{\prime }$ and so on; (d) This process is repeated in all classes of the problem. For example, a problem with two classes results in the reference set $REF=\{P_{e},P_{A},P_{B},...,P_{N}\}$.

In addition to the measures of entropy, divergence, and complexity generated from the uniform histogram $P_{e}$, for each new class, there will be an additional measure of divergence and complexity. For example, in the problem of two classes, class A and class B, with their respective histograms of references $P_{A}$ and $P_{B}$, the values of the measure of divergence can be obtained by the formulas of Table 1.

The process ends when all the reference histograms are used to calculate the divergence and complexity measures. The set of characteristics obtained for a multiclass problem is similar to that shown in Table 2.

In the example of Table 2, the complexity values are calculated based on three classes. The complexity values will decrease for a specific class, for example, the class *A*, if the reference histogram used is related to class *A*. This happens because the calculation of divergence between the histograms that belong to the same class results in lower complexity values. These differences are caused by each reference histogram, although small, are fundamental for the learning methods to find patterns and generate classification models with good performance. This methodology also results in a reduced number of features for learning algorithms.

### 4.4. Classification Model

The problem addressed in this research is a classification problem. Several classification methods can be used, such as decision tree, neural networks, SVM, naive Bayes, among others. However, this work has chosen the KNN method to facilitate the comparison and evaluation of the HAR-SR with other similar works in the literature. As a measure of KNN distance, the similarity of cosines commonly used by classification methods based on symbolic representation.

The classification process consists of associating a label of an existing class with a new time series $T_{u}$ as yet unlabelled. For this task, the model goes through two steps:

1. The training
  (a) A time series dataset $D=\{\left(T_{1},y_{1}\right),\left(T_{2},y_{2}\right),\ldots ,\left(T_{N},y_{k}\right)\}$ is an unordered set of *N* time series, *k* is the number of classes, $T_{i}$ is the time series, and $y_{i}$ is the label associated with the series $T_{i}$;
  (b) Creation of a reference histogram for each of the classes. For example, for two classes, $REF=\{P_{e},P_{A},P_{B}\}$, where $P_{e}$ is the uniform histogram and $P_{A}$ and $P_{B}$ are the reference histogram for class *A* and class *B*, respectively;
  (c) For each time series $T_{i}$, apply the segmentation $S\left(a,w\right)=\left(t_{a},...,t_{a+w-1}\right)$, with 1≤a≤n−w+1, the data fusion, perform the discretization to obtain the histogram $P_{i}$;
  (d) Calculation of statistical measures using each reference histogram.
  (e) Generation of the training set from the statistical measures.
2. The classification
  (a) Given a new series not labeled $T_{u}$;
  (b) Apply the segmentation, the data fusion, and the discretization to obtain the histogram $P_{u}$;
  (c) Calculate the statistical measures that use the reference histograms obtained in the training phase, resulting in the feature vector of the $T_{u}$ series;
  (d) The label associated with the series $T_{i}\in D$ closest to the $T_{u}$ series will be assigned to the uncollected series $T_{u}$, using the cosine similarity metric.

**Figure 9:**
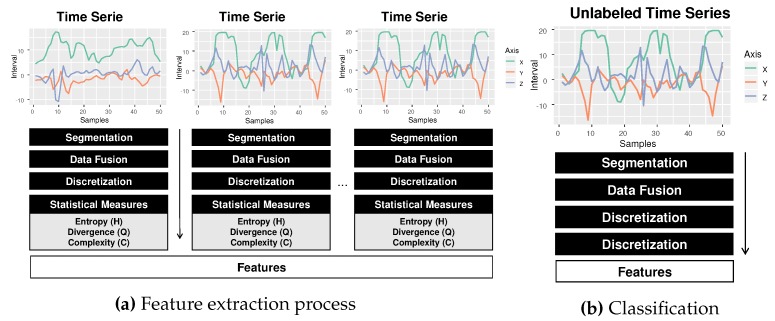
(**a**) The feature extraction process adopted by the HAR-SR method. (**b**) To classify a new non-labeled series *T_u_*, the procedure is similar to that performed in the training phase. The difference is that there is no need to obtain new reference histograms.

Figure 9 shows an example of the feature extraction process in the training phase for a multiclass problem. To classify a new non-labeled series $T_{u}$, the procedure is similar to that performed in the training phase. The difference is that there is no need to obtain new reference histograms. The process of extraction of characteristics is shown in Figure 9b. Finally, the label associated with the nearest series will be assigned to the non-labeled series using a cosine similarity metric, using Equation (7). With the new classified activity, the classification process is finalized.

(7) $$arg_{max}=\left\{similarity\left(T_{i},T_{u}\right)=\frac{T_{i}.T_{u}}{∥T_{i}∥.∥T_{u}∥}\right\},$$

where $T_{i}$ belongs to the training set and $T_{u}$ the non-labeled series.

### 4.5. Computational Analysis

The SAX algorithm is based on average calculations and therefore its implementation is very simple. The approximation process uses the PAA algorithm to divide the series *T* into ω parts. The quantization process consists of a search in the quantization table β, which is fixed and predefined for all domains. In this way, the complexity of the SAX is dominated by the complexity of the PAA algorithm, as shown in Equation (8), which is linear in *n* [19,21,22]:

(8) $$SAX\left(T_{n}\right)=O\left(n\right)$$

The SFA approach process is dominated by the computational complexity of a single DFT for a $T_{n}$ series, resulting in complexity Olog(n). If the sliding window technique of size *w* is used in the $T_{n}$ series, the complexity of a subsequence using DFT will be Owlog(w), and for all n−w+1 windows it will have computational complexity O(nwlog(w)). However, optimizations in the SFA algorithm allow the MFT to be used to deal with the problem of significant overlap of the windows *w* calculations. In this way, the DFT is applied only in the first window O(wlog(w)) and all other windows (n−w) are calculated through the MFT O(ω). Thus, the new computational complexity of the method is reduced according to Equation (9):

(9) $$MFT\left(T_{sw}\right)=O\left(\omega \left(n-w\right)+w\log\left(w\right)\right)$$

For the set *D* of size *N*, the computational complexity is O(N(ωn+wlog(w)). The application of the MFT throughout the set completes the approach process. Subsequently, the quantization process consists of using the MCB table as a search table, which contains quantization intervals. First, the set *D* is used, represented by an array containing all *N* Fourier transformations of size ω. Then, given this array, the columns are ordered and partitioned at evenly spaced intervals. The ordering of ω columns with *N* values has complexity O(ωNlog(N)), using merge sort, for example. The equidistant intervals applied to the ordered columns require a search on each column, adding a complexity of O(ωN). Thus, the total complexity of the process of approximation and the creation of the MCB table is defined by $sort\left(FT_{n}\right)+quantization\left(FT_{n}\right)+SFA\left(FT_{n}\right)$ according to Equation (10). The complexity of DFT and MFT are shown in Figures 11 and 12.

(10) MCB(FT)∈O(ωNlog(N))+O(ωN))+O(Nnlog(n))

(11) DFT(FT)=O(N(ωlog(N)+ω+nlog(n)))

(12) MFT(FT)=O(N(ωlog(N)+ω+n)+wlog(w))

The result of the computational complexity shows that the discretization step (calculation of the MCB table) of the preprocessing phase is dominated by the *N*-size Fourier transform and the ordering to obtain the equidistant intervals.

The transformation phase of the SFA, which takes place after the preprocessing phase, consists of the discretization of a *T* series using the MCB table. Then, to transform a *T* series using the MFT, an SFA word of size ω requires ω searches in the MCB table containing α ranges. This results in O(ωlog(α) O) operations (using binary search). Thus, the total complexity in the process of transforming the SFA into a *T* series, given the MCB table is:

(13) SFA(T)∈SFA(T)+search(T).

The HAR-SR works similarly to the BOP model and the BOSS model. These (structure-based) models extract a vector of high-level characteristics to construct a classification model from learning algorithms such as KNN, decision tree and SVM. The process of extracting substructures is simple. Applies to the sliding window technique in a time series *T* extract *w* subsequences. Then each subsequence is discretized through SAX or SFA, whose complexities are already known. Therefore, the complexity of the HAR-SR to extract substructures depends on the discretization method used.

In both cases, to transform a series T using the search table β, a word of size ω requires ω searches containing α ranges. This results in O(ωlog(α)) operations (using binary search). Thus, it is possible to calculate the complexity adopted in the HAR-SR method if the discretization algorithm is the SAX or SFA through the Table 3:

The statistical measures are used as new features obtained from the histogram $P_{\eta }$, where η is the size of the sample space. The calculation of entropy, can be calculated in linear time in η, and thus, its computational complexity is defined in Equation (14):

(14) H(P)=O(η)

The divergence can be calculated in linear time in η, and as the entropy had already been calculated previously, its computational complexity is defined in Equation (15).

(15) Q(P)=O(η)+O(η)=O(η)

The HAR-SR classification process consists of associating an unlabelled $T_{u}$ series using a training set. For this task, the HAR-SR uses the KNN algorithm to perform a search between the new $T_{u}$ series and the DS training set whose objective is to find the label for the $T_{u}$ series. Then, the series $T_{u}$ is associated with the class of the series *T* that minimizes the value of the distance metric adopted

(16) $$label\left(T_{u}\right)=argmin_{T\in DS}\left(T_{u},T\right)$$

This procedure has complexity O(N(k+n)), where *N* is the size of the training set, *n* is the size of each instance of the set DS, and *k* is the parameter of the KNN. Each distance calculation requires O(n) runs, and the cost of this calculation over the entire set DS is O(Nn). To select the *k* nearest values, more O(kn) are required.

## 5. Experimental Protocol

This section presents the experiments used to evaluate the HAR-SR method in human activity recognition. We compared the proposed method with different methods found in the literature in different scenarios. The performance metric used was to measure F1 and accuracy. An important step in this process is the use of leave-one-subject-out cross-validation (LOSO) as a validation procedure applied in the datasets. This step is important as it influences the overall performance of the HAR-SR. Finally, the method was compared with several related works and analyzes, and discussions about the results are performed.

### 5.1. Datasets

#### 5.1.1. SHOAIB Dataset

The first dataset (SH) was proposed by Shoaib et al. [47]. There were ten participants involved in our data collection experiment, all male, between the ages of 25 and 30. These were walking, running, sitting, standing, jogging, biking, walking upstairs and walking downstairs, which are mainly used in the HAR related studies, and they are the basic motion activities in daily life. Each volunteer performed eight activities for 3 to 4 min for each activity. The activities used were walking, running, sitting, standing, jogging, upstairs, and downstairs. The experiments were carried out indoors in one of the university buildings, except biking. For walking and running, the department corridor was used. For walking upstairs and downstairs, a 5-floor building with stairs was used. Each of these participants was equipped with five smartphones on five body positions. Data were collected from a smartphone using the accelerometer, linear accelerometer, gyroscope, and magnetometer sensors at a frequency of 50 Hz.

The selection of this dataset was motivated by the following aspects: (a) the SH dataset contains data collected at five different body positions: right jeans pocket, left jeans pocket, belt position towards the right leg using a belt clip, right upper arm, and right wrist. This allows the evaluation of different scenarios of activity recognition depending on the smartphone position; (b) it contains six activities with data collected from accelerometer, gyroscope and magnetometer sensors, which are mainly used in the HAR studies; (c) the dataset is balanced and should represent a fairer evaluation, which means a reduction in bias caused by individuals with more activity data and individuals with more activity labels than others.

#### 5.1.2. WISDM Dataset

We have also evaluated our method on the WISDM dataset, proposed by Kwapisz et al. [31]. The data collection was controlled by an application through a simple graphical user interface, which allowed recording the user’s name, starting and stopping the data collection, and labeling the activity being performed. It also allows controlling what sensor data (e.g., GPS, accelerometer) was collected and how frequently it was collected. In all cases, the accelerometer data were collected every 50 ms, or 20 samples per second (20 Hz). The data collection was supervised by one of the WISDM team members to ensure the quality of the data. Data were collected from 36 volunteers performing six physical activities: walking, jogging, walking downstairs, walking upstairs, sitting, and standing.

#### 5.1.3. UCI Dataset

The last dataset is the UCI dataset, proposed by Anguita et al. [9], which contains data from 30 volunteers aged 19 to 48 years. Each volunteer was instructed to follow a protocol of activities while wearing a waist-mounted smartphone. In the first test the smartphone was attached to the left side of the belt and in the second test, the participant could choose a place of their choice. There is also a separation of 5 seconds between each task where individuals are told to rest. This procedure facilitates the repeatability and ground trough generation through the visual interface. The six activities performed were walking, walking upstairs, walking downstairs, sitting, standing, and lying down. The sensors used in the experiment was the accelerometer and gyroscope at a rate of 50 Hz. This rate is sufficient for capturing human body motion since 99% of its energy is contained below 15 Hz.

#### 5.1.4. Summarization of Datasets

Table 4 summarizes the characteristics of the datasets used in the experiments. In our experiments, for the WISDM dataset, we only selected the users that had instances of all six activity classes, totaling 19 individuals. For the SHOAIB dataset, we selected the six most similar activities with WISDM and UCI-HAR dataset, so that all datasets had the same number of classes to compare results. For this reason, the Jogging and Biking activities (marked with * in Table 4) were removed from our experiments. The sensors of all datasets, e.g., accelerometer and gyroscope, were selected according to the evaluation scenario. In a given scenario all sensors were used, in others, only the accelerometer sensor was selected, and so on.

### 5.2. Baselines

The baselines used for comparison included:

(a) a shallow approach based on hand-crafted feature extraction from the time and frequency domain of the signal, called TF. The list of the mathematical functions used to create the feature set is found in Table 5;
(b) a discrete domain classification method, SAX-VSM. SAX-VSM uses a technique called tf-idf in the frequency histogram symbols of each activity class to obtain a weighted frequency matrix. The result is an array that contains an instance for each activity and will be the feature set used by the classification model;
(c) a discrete domain classification method, BOSS-VS classifier. Like SAX-VSM, the BOSS-VS uses tf-idf in the frequency histogram to obtain a weighted frequency matrix for each activity. The parameters used in the comparison of the methods are shown in Table 6.

For symbolic representation algorithms, which have parameters such as word, alphabet, segment size, and window size, the parameters chosen was with values attributed to ω=6 and α=6 based on several experiments performed in the SHOAIB database because it has more diversity between the position parameters and sensors. As demonstrated by Lin et al. [19] and Schäfer [37], the complexity of these methods may evolve as the value of these variables increases. For example, with a sample space of size 256 it is possible to generate a less complex model. However, the sample space of 46,656 may be necessary and more representative when more complex problems need to be solved.

### 5.3. Validation Procedures

Most studies in HAR use k-fold cross-validation (k-CV) as the default validation procedure to validate the machine learning model performance [48]. To build a universal method in scenarios with a group of individuals, the k-CV may not be the best solution because the segments belonging to a certain individual can be present in both the training set and the test set. In the HAR context, this means that the model trained using k-CV may know the activity patterns of a specific individual that is present in the test set, obtaining higher accuracy in the classification. In this scenario, this situation may lead to overfitting problem. To minimize this problem, this work adopts the leave-one-subject-out cross-validation (LOSO) as the default validation procedure. The LOSO considers the individual information when split the training and test set, preventing that some data from the same individual to be present in both sets [49]. The results presented in Ignatov, Lockhart and Weiss show that classifiers tend to lose performance when using the LOSO instead of k-CV validation procedure. The higher accuracy methods that use traditional k-CV do not always have good results in a real domain because they are over-adjusted for the set in which they were trained. The LOSO procedure is also applied by HAR-SR to generate the reference histograms. In this context, none of the histograms used to train the model contain information about the test set.

### 5.4. Scenarios

We evaluated the performance of the proposed method on four evaluation scenarios:

(a) Scenario A evaluated the parameters used by the discretization methods. The symbolic representation algorithms (SAX and SFA) had parameters such as the word, alphabet, and window sizes. The complexity of these methods could evolve as the value of these variables grew.
(b) Scenario B evaluated data fusion techniques, magnitude, PCA, and signal concatenation. The data fusion techniques played an important role: providing the highest quality signal compared to each signal individually. This study is important because the complex calculation of the algorithms that make up the SAX and SFA are calculated based on the input data. In this case, it was necessary for the chosen technique to transform a multidimensional signal into a one-dimensional signal, preserving the characteristics of the signals;
(c) Scenario C evaluated the methods of symbolic representation by the position of the smartphone. This scenario was useful for showing the differences between representations and their impact on rating method performance. Four groups of different sensors (SG1, SG2, SG3, SG4) were used to train and test the classification method. The idea was to show what the impact was on the performance of the classification method when adding a new sensor. Another result obtained in this scenario shows how each sensor group behaved according to the positions in which the smartphone was located;
(d) Scenario D evaluated the performance of the proposed method HAR-SR with three works in the literature. The first work, called TF method, used a total of 145 hand-crafted features belonging to the time and frequency domain. The last two other works were feature learning approaches of the discrete domain, similar to the proposed method.

## 6. Results

### 6.1. Scenario A: Parameter Evaluation

**Figure 10:**
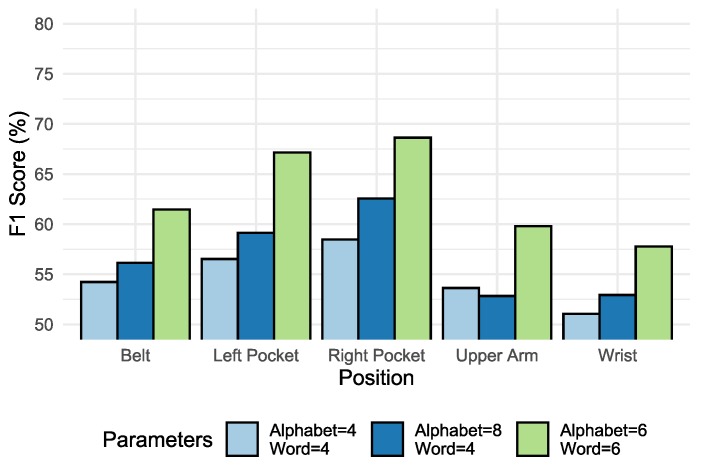
Average performance of symbolic algorithms with variation in parameters ω and α for each body position of SHOAIB dataset.

The experiments performed to select the best parameter set show that for the range of values defined in Table 7, the best results were those whose value was assigned to ω=6 and α=6. This study was performed in the SHOAIB database because it had more diversity between the position parameters and sensors. The mean of the results for each position can be seen in Figure 10, which presents the best parameters whose value was attributed to ω=6 and α=6. For this reason, the comparison of the results presented in this section adopts by default ω=6 and α=6.

Table 8 shows the growth of the sample space according to the word and alphabet size values. For performance reasons, this work limits the maximum value assigned to ω=6 and α=6. For example, with a word space of size 256, it is possible to generate a very simple model in terms of computational complexity, however, the word space of 46,656 may be necessary and more representative when more complex problems need to be solved.

### 6.2. Scenario B: Data Fusion

This section aims to evaluate three data fusion techniques: magnitude, PCA, and signal concatenation. The SHOAIB database was selected because it has data from three sensors (accelerometer, gyroscope, and magnetometer). In addition, it has a variety of positions, thus allowing many scenarios to be analyzed. The sensors available for testing were grouped and evaluated at each available position in the base. Table 9 shows the sensors arrangement for evaluating scenario *A*.

**Figure 11:**
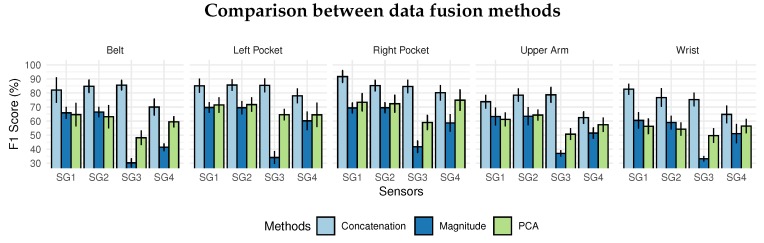
Comparison between data fusion techniques. The dataset used was SHOAIB. The discretization technique was SAX, with segmentation of 2.5 s, ω=6 and α=6.

The results presented in Figure 11 are based on the average of LOSO results from the five-position data of the SHOAIB dataset: belt, left pocket, the right pocket, arm, and wrist. The concatenation technique presented the best results with 82% overall, with 73.77% for wrist position as the worst result. The magnitude and PCA presented low performance, because the dimensionality reduction applied in the signals, which implied a loss of information about the signals.

The comparison between magnitude and PCA were similar for the group SG1 and SG2. However, magnitude presented the worst results in the SG3 group when the magnetometer sensor was inserted with 41.78% in the best result. Using only the gyroscope (SG4), the PCA presented, on average, 60% in F1-score against 50% for magnitude with a 10% difference.

The results show that the accelerometer was the most suitable sensor for recognizing activities in all positions, confirming results obtained in previous works. The addition or combination of new sensors such as the gyroscope and magnetometer on average presented a gain on performance no more than 5%, considering the margin of error, except in some cases when the inclusion of new sensors began to degrade the performance of the method, as shown in Figure 10. The addition of new sensors meant more cost of processing and more memory usage, which would only be relevant if the impact on classification were noticeable.

### 6.3. Scenario C: Evaluation of Symbolic Algorithms by Position

**Figure 12:**
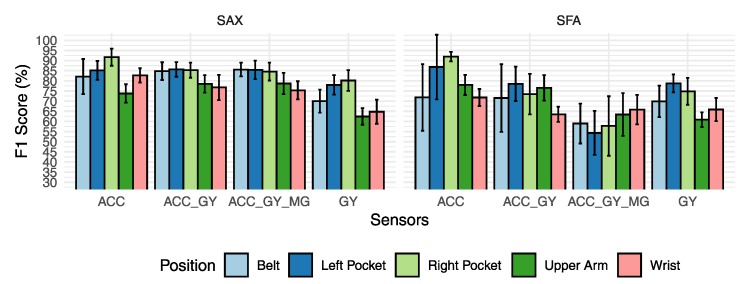
Classification performance at each position of SHOAIB dataset. The discretization method used in this experiment was SAX and SFA, with T=2.5 s, α=6, ω=6.

This scenario will be useful to show the differences between the representations and their impact on the overall performance. It shows how each sensor group behaves according to the positions in which it is located on the smartphone. For this scenario, only concatenation was used as a fusion method. The results presented in Figure 12 show some difference in classification performance for the discretization algorithms with different behaviors according to the choice of the sensors. The results of the SAX algorithm show that there was only some gain classification performance by adding new sensors to recognize activities in the belt and arm position.

The results for the SFA method show a drop in classification performance when new sensors were introduced. The performance loss achieved 18% on the worst scenario for the right pocket. The SAX algorithm, when using only the accelerometer sensor, had similar performance for the left and right pocket positions with 86% and 91%, better performance for the arm position, and for arm position exhibited worse classification performance with 73.77% against 78.01% for SFA. The conclusion of the results presented in Figure 12 is that SAX presented the best results in most of the evaluated positions.

The results of the comparison between SAX and SFA are interesting because they show that in this context of activity recognition, the SAX algorithm could extract patterns that were more general for the whole set of individuals. In other domains, as shown in Schäfer and Bagnall et al., SFA presented better results.

One of the possible reasons for these results is based on the creation of the MCB table, which is responsible for determining the breakpoints according to the variables α and ω. The construction of the MCB table is supervised and depends on the quality of the data. In this context, if the table is created using only data from similar individuals, for example, it should produce a table that represents that specific group very well. However, it should not perform well when individuals with particularities in their activity patterns are entered into the test. This training process of the MCB table is dependent on the training database. This influences the less representative discretization process for a more generic group of individuals. The SAX algorithm uses a fixed breakpoint table, with values based on a Gaussian distribution. For this reason, the generalization capability of the model must be greater than in a model that takes information only from the knowledge of a limited dataset.

### 6.4. Scenario D: Comparison of HAR-SR with Other Studies

This scenario compared the classification performance of works found in the literature with the HAR-SR method proposed in this paper. In this scenario, only the accelerometer sensor was used, and the parameters chosen for the discrete methods were SAX as a discretization algorithm, with the segmentation of T=2.5 s, α=6 and ω=6.

**Figure 13:**
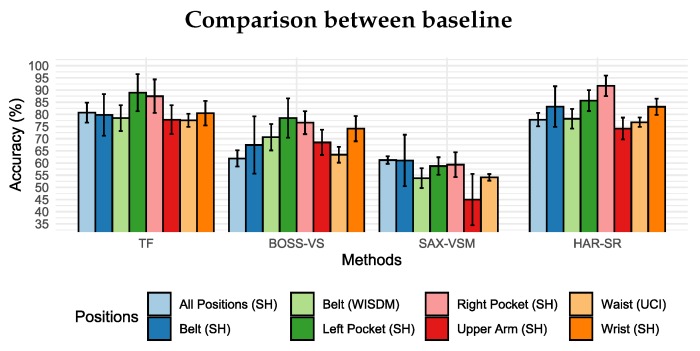
The comparison between the TF, SAX-VSM, BOSS-VS methods and the proposed HAR-SR method. Only the accelerometer sensor was used.

Compared with the BOSS-VS model and SAX-VSM model, the HAR-SR method presented the best results in all positions analyzed. Figure 13 shows that HAR-SR outperformed SAX-VSM with more than 80% against 55% for SAX-VSM. Besides, the performance difference between the BOSS-VS and SAX-VSM was already expected, as presented in other studies [23].

**Figure 14:**
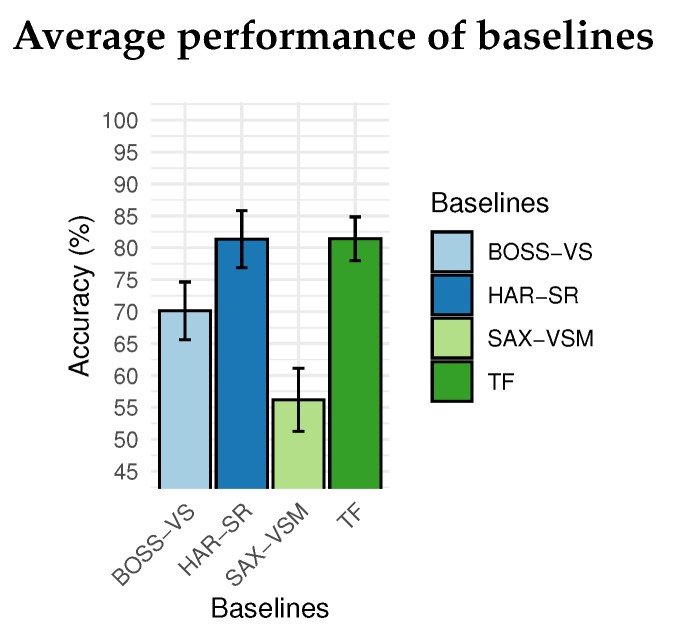
Average performance in E1 scenario.

Figure 14 shows that the TF method and the HAR-SR method had similar performance in the classification of activity in all positions, both with 81% on average, considered a technical tie given the confidence margin. This shows, at first, that the HAR-SR method had the same capabilities as the TF method, but used a smaller number of features. For this reason, the confusion matrix will be used in the next section to indicate which classes the algorithms have more difficulties in classification. This analysis should show the particularities of these two methods and how new problems can be addressed in the future.

#### Confusion Matrix Analysis

**Figure 15:**
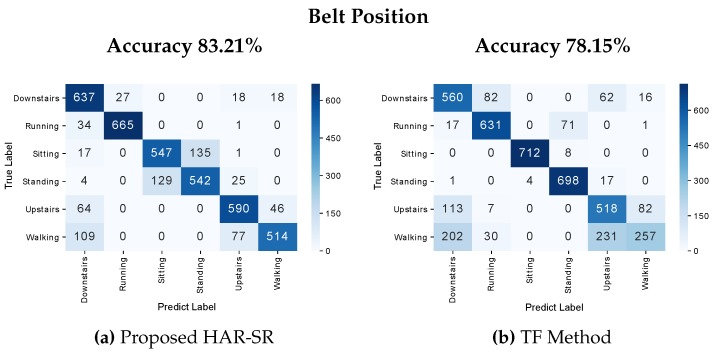
Confusion matrix for accelerometer sensor at belt position.

The results presented in this section are based on the SHOAIB database, for belt position and position-independent. At the belt position, the HAR-SR method performed with 83.21%, with classification errors mainly in stationary activities (e.g., standing or sitting), as shown in Figure 15. Another type of behavior was observed in the TF method, which found problems in differentiating non-stationary activities (e.g., downstairs and upstairs).

**Figure 16:**
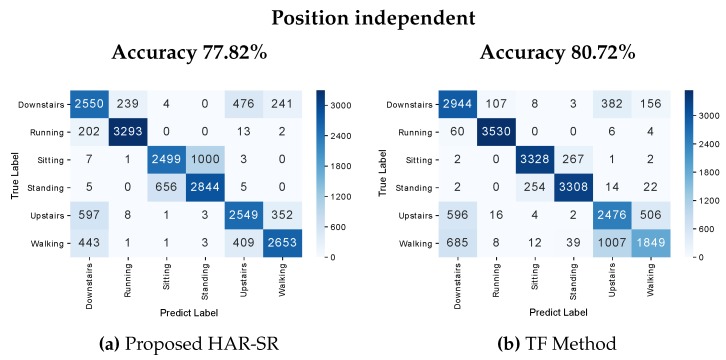
Confusion matrix for acclerometer sensor at all positions (independent).

The confusion matrix presented in Figure 16 was position independent; that is, the position labels were removed from the dataset, and the methods had to find the different types of patterns for each position. This additional work that classification algorithms had to recognize patterns of activities where there was no explicit information about the position is a scenario compatible with what happens in the real world. The HAR-SR method achieved 77.82% against 80.72% for the TF method. The HAR-SR method, in general, had more difficulty in classifying between stationary activities. However, it presented good results in the classification of non-stationary activities.

### 6.5. Discussion

To create a low-cost solution for HAR, the first way investigated in this research was by reducing the data dimensionality from input data. The first evaluation was performed aiming the best symbolic representation parameters for build the classification model since the discretization process is a critical phase of our method. The results have shown that choosing larger or smaller parameters will adapt the degree of approximation of the algorithm for the original signal. With smaller word space generates very simple models in terms of computational complexity, however, they are not suitable for complex problems, and tends to lose performance. The best results obtained are those whose value was assigned to ω=6 and α=6.

Another conclusion of this study is that the quality of the symbolic representation of a time series depends on the quality of the signals obtained in the data fusion process. The magnitude, PCA, and simple concatenation techniques transform the sensor signals into a one-dimensional form since the data collected from the motion sensors are multi-dimensional. This process is fundamental to reduce the overall complexity of the algorithm, and at the same time enable the use of symbolic representation methods, such as SAX and SFA.

The results have shown that the use of magnitude and PCA is insufficient to generate a representative signal for physical activities. These methods resulted in poor classification performance, which mostly did not exceed 60% in F1-Score. A possible explanation is that both magnitude and PCA have a lot of data loss during the data fusion process. The simple concatenation method presents the best results in all scenarios, although it is not a data fusion method itself, and for this reason, the discretization algorithm will have a larger segment containing all the data collected.

After applying data fusion in the input signal, we evaluate the symbolic representation methods. The dimensionality reduction is intrinsic to symbolic representation methods. These methods transform the data represented by real values into a discrete domain. This allows the application of several data mining techniques methods, such as the tf-idf model used by the SAX-VSM and BOSS-VS.

The SAX algorithm obtained the best results in all scenarios. The SAX is simpler than SFA because of its approximation process which uses a pre-defined search table. The SFA has an extra step to generate the search table, called MCB, which adds an extra computational cost to compute the search table. The reason for the best results obtained by SAX is because the SFA tends to overfit its MCB table based on the training database. If the table is created using only data from similar individuals, for example, it should produce a search table that represents that group of individuals very well. This influences the less representative discretization process for a more generic group of individuals, or it should not perform well when individuals with particularities in their activity patterns are entered into the test. The SAX algorithm uses a fixed breakpoint table, with values based on a Gaussian distribution. For this reason, the generalization capability of the model must be greater than in a model that takes information only from the knowledge of a limited dataset.

An important finding in this research is that the position where the smartphone is located is related to the number of sensors required for a good performance of the classification method. This analysis is important so that in future research, the selection of sensors is performed according to the position of the smartphone in the individual. The results have shown that the discretization algorithms present different behaviors according to the choice of the sensors. For example, the results have shown that there is some gain in method performance by adding new sensors to recognize activities in the waist and arm position. In the positions of the left pocket there is no gain and for the positions of the right pocket and pulse occurs a loss in the performance of the method. In addition, in the left and right pocket positions, the gyroscope sensor performs well.

Some activities have the same results when a new sensor is added. In some cases, the difference is very small, about 2%, which makes it unfeasible to use the sensor. Besides, a new sensor can add unnecessary processing to the method, increasing its complexity, and can also result in a loss of performance in the classification. For example, in the positions of the left pocket there is no gain and for the positions of the right pocket and pulse occurs a loss in the performance of the method. The overall results have shown that the accelerometer is the best sensor for activity recognition.

The HAR-SR method outperforms SAX-VSM and BOSS-VS in the HAR domain. In comparison with the TF method, which uses time and frequency features, the HAR-SR results are similar with no statistical difference considering the confidence margin. The HAR-SR also generates a smaller number of features when comparing TF, BOP-SAX, and BOSS methods. This can be an advantage, mainly because the classification model built is simpler. A negative characteristic of HAR-SR is the search space for ideal parameters used by symbolic algorithms. Choosing incorrect values for window, word and alphabet size can result in data representation loss, and this may impact directly in the classification performance.

The use of LOOCV methodology has shown interesting results. First, a decrease in the classification performance for the community methods, which has high levels of accuracy (above 90%). The results have shown that classifiers tend to lose performance when using the LOSO validation metric. In the HAR domain, the use of traditional cross-validation may not be a good way to represent the real world, and consequently, the models obtained may not reflect reality. The use of LOOCV is important because it is the indicator of the generalization potential of the application, without the need to obtain data from the individual before using the system. However, high levels of accuracy using traditional CV may be a good indication of personalization for the classification model, which can extract well the data standards known by the model. This enables the creation of personalized models that capture the characteristics of each user.

## 7. Related Works

This section presents a summary of the main works in HAR that use inertial sensors of smartphones.

Kwapisz et al. [31] provide the WISDM dataset, containing 36-user accelerometer information, which is used in many RAH works. The results show an accuracy of 91.7% for neural networks using shallow features in the time domain. Anguita et al. [9] provide the UCI-HAR dataset, which contains data from the accelerometer and gyroscope sensors extracted from 30 users who performed six physical activities. In total, 561 features were extracted in the time and frequency domain. The results have shown an accuracy of 96% using SVM. Shoaib et al. [47] provide a dataset with eight physical activities collected from four different body positions: belt, left pocket, the right pocket, arm, and wrist. The best result is represented by the 98% accuracy when the smartphone is in the belt position.

Micucci et al. [50] provide a public dataset for the RAH domain, which contains physical activity data and data representing falls. To recognize the nine types of physical activity, 453 features of the accelerometer signal were extracted, and four classifiers were used: the KNN, SVM, ANN, and Random Forest. In the best scenario, its method obtained 88% accuracy using the Random Forest method (five-fold CV) and 73% using the random forest method with the Leave-one-subject-out cross-validation (LOSO) metric.

For the symbolic domain, one of the first works that uses SAX to recognize activities based on smartphones is described by Figo et al. [7]. However, the best outcome in terms of accuracy was 50.5% for three activities (running, walking, and jumping). The second scenario consisted of two activities (walking and running) and presented an accuracy of 84.17% using the Dynamic Time Warp (DTW). The authors have shown that the discrete domain features are feasible for applications in mobile devices. The investigation of preprocessing techniques to improve the results in a scenario with more than two activities is given as future work.

Siirtola et al. [51] presented Symbolic Aggregate Approximation Similarity (SAXS), which is based on the similarity calculation between words obtained through the SAX algorithm. The authors also use a reference model for each activity, so that the similarity between the reference model and a new activity instance is calculated. The SAXS was evaluated in five databases in different contexts. The results showed an accuracy of 83.8% in swimming activity, 84.3% in gestures, 85.7% and 82.96% in tool use, and 83.26% in sports.

Akimura et al. [52] propose the use of a lightweight compression technique to minimize the size of the activity sensor data sent over a wireless link. The compressed sensing (CS) technique consists of very simple matrix operations at the mobile side, and CPU-intensive reconstruction is performed on the resource-rich machine on the network side. The method was evaluated by using activity data consisting of six activities. The presented results shown a reduction in power consumption by approximately 16% as compared with traditional ZIP compression, while keeping the reconstruction error to less than 10% and the recognition accuracy of the six basic activities over 70%.

Lima et al. [36] present a comparative analysis of the symbolic representation algorithms in HAR domain, such as SAX-VSM, BOSS MODEL, BOSS-VS and WEASEL, and the traditional shallow features in time and frequency domain. The results have shown that the symbolic representation algorithms are more accurate than the shallow algorithms, obtaining approximately 99% accuracy when the validation metric used is the 10-fold CV. In addition, they are on average 84.81% faster in the feature extraction step, and that they reduce on average 94.48% of the memory space consumption.

Quispe et al. [53] present an approach for HAR based on symbolic data representation of time series. The Bag-Of-SFA-Symbols (BOSS) method is extended to multi-dimensional time series. The proposed method MBOSS adopts a data fusion approach at the level of features extending the BOSS model. It uses a histogram fusion process, concatenating the *M* characteristics (size of the histogram) of dimension *N* (number of variables) into a single vector of characteristics of dimension MxN. The final characteristic vector, called the MBOSS model, it is used for the classification task. Experiments on accelerometer data from three publicly datasets have shown that the MBOSS achieve 99.37% in subject-dependent evaluation and 87.49% in subject-independent evaluation in the best scenario.

Hur et al. [15] propose an efficient human activity recognition method, namely Iss2Image (Inertial sensor signal to Image), an encoding technique for transforming an inertial sensor signal into an image with minimum distortion and a convolutional neural network (CNN) model for image-based activity classification. The Iss2Image converts real number values from the *X*, *Y*, and *Z* axes into three color channels to precisely infer correlations among successive sensor signal values in three different dimensions. The results have shown that the Iss2Image presents higher accuracy than other state-of-the-art approaches, achieving 100% in MobiAct dataset, 97.11% in UCI-HAR and 100% in UC-HAR.

Gani et al. [54] present a lightweight smartphone method for human activity recognition based on dynamical systems and chaos theory. The method is an alternative approach to widely used machine learning techniques to recognize human activities. A reconstructed phase space is formed from the accelerometer sensor data using time-delay embedding. A Gaussian mixture model is learned on the reconstructed phase space. A maximum likelihood classifier uses the Gaussian mixture model to classify ten different human activities. The proposed approach achieved 100% accuracy for individual models across all activities and accuracy of 90% to classify six different activities of 30 participants.

Chung et al. [16] develop a Long Short-Term Memory (LSTM) network framework to support training of a deep learning model on human activity data. The proposed method adopts a two-level ensemble model to combine class-probabilities of multiple sensor modalities, and demonstrate that a classifier-level sensor fusion technique can improve the classification performance. The results indicate that applying sensor data from additional modalities can contribute to improving the recognition accuracy of nine activities up to 93.07%. In addition, soft-voting achieves higher overall performance (94.47%) than hard-voting (93.48%), because it can give more weight to highly confident votes.

In general, the works presented in the literature based on shallow features always uses the same set of features, with some variations. For the classification task, it is common the use of a framework, called the WEKA tool [34], which aggregates preprocessing techniques and machine learning methods. Our approach is a lightweight version of the approaches that uses the tf-idf model, e.g., SAX-SVM model, BOSS model, and MBOSS model. In these approaches, the tf-idf model is applied over the frequency distribution generated by the discretization process. The result of this process is a weighted frequency distribution that is used as features by machine learning algorithms. Alternatively, our method uses information theory quantifiers as new features extracted from a frequency distribution, but the resulting feature set is significantly smaller.

## 8. Conclusions

In this paper we have shown that HAR-SR presents a solution for human activity recognition with low computational cost, being inferior to the quadratic order $O(n^{2})$, making it feasible for implementation in smartphones with limited resources.

The results obtained from four evaluation scenarios were used to observe the behavior of different stages of the process of recognizing human activities. The fusion techniques magnitude and PCA were insufficient to generate a representative signal for physical activities, presenting a lot of data loss. These methods resulted in poor classification performance, which mostly did not exceed 60% in F1-Score, which makes it unsuitable for our method.

The symbolic representation algorithms are critical in our model, and the parameters chosen will impact directly on the recognition performance. We have found an intermediate, cost-benefit set of parameters. Larger parameters are too expensive in terms of computational complexity and they are not suitable for mobile devices. The SAX algorithm presents the best performance over the SFA algorithm in most scenarios. In addition, the discretization algorithms have shown different behaviors according to the choice of the sensors. For some activities, there is no difference in the results when a new sensor is added. In some cases, the difference is very small, about 2%, which makes it unfeasible to use the sensor. This result has shown that a new sensor can add unnecessary processing to the method, increasing its complexity, and can also result in a loss of performance in the classification by add unnecessary noise. The overall results have shown that the accelerometer is the best sensor for activity recognition.

We have shown the HAR-SR method outperforms SAX-VSM and BOSS-VS in the HAR domain. In comparison with the TF method, which uses time and frequency features, the HAR-SR results are very similar but generate a smaller number of features when comparing TF, BOP-SAX, and BOSS methods. A negative characteristic of HAR-SR is the search space for ideal parameters used by symbolic algorithms. Choosing incorrect values for window, word and alphabet size can result in data representation loss, and this may impact directly in the activity recognition performance.

Future directions involve the study of new data fusion techniques that aggregate new information to discriminate characteristic signals from stationary activities. This may improve recognition performance, making the method even more robust.

## Figures and Tables

**Table 1:** Calculation of the divergence based on *N* classes.

Divergence	Complexity
$Q_{A}\left[P,P_{A}\right]=Q_{0}H\left[P,P_{A}\right]=Q_{0}H\left\{\begin{array}{c} \left[\frac{P+P_{A}}{2}\right]-\frac{H[P]}{2}-\frac{H[P_{A}]}{2} \end{array}\right\}$	$C\left[P\right]=Q_{j}\left[P,P_{A}\right]H\left[P\right]$
$Q_{B}\left[P,P_{B}\right]=Q_{0}H\left[P,P_{B}\right]=Q_{0}H\left\{\begin{array}{c} \left[\frac{P+P_{B}}{2}\right]-\frac{H[P]}{2}-\frac{H[P_{B}]}{2} \end{array}\right\}$	$C\left[P\right]=Q_{j}\left[P,P_{B}\right]H\left[P\right]$
...	...
$Q_{N}\left[P,P_{N}\right]=Q_{0}H\left[P,P_{N}\right]=Q_{0}H\left\{\begin{array}{c} \left[\frac{P+P_{N}}{2}\right]-\frac{H[P]}{2}-\frac{H[P_{N}]}{2} \end{array}\right\}$	$C\left[P\right]=Q_{j}\left[P,P_{N}\right]H\left[P\right]$

**Table 2:** Table of values obtained by calculating the entropy, divergence and complexity for a problem with multiclasses.

$H_{s}$	$Q_{\mathrm{ref}}$	$Q_{A}$	$Q_{B}$	…	$Q_{N}$	$C_{\mathrm{ref}}$	$C_{A}$	$C_{B}$	…	$C_{N}$	Class
0.54	0.07	0.10	0.06	…	0.06	0.04	0.05	0.03	…	0.03	A
0.49	0.08	0.06	0.08	…	0.02	0.04	0.03	0.10	…	0.02	B
…	…	…	…	…	…	…	…	…	…	…	…
0.49	0.08	0.12	0.10	…	0.01	0.04	0.05	0.15	…	0.03	A

**Table 3:** Complexity of Bag-of-patterns using SAX and SFA algorithms.

Method	Complexity
$SAX(FT_{sw})$	O(Nnωlog(α))
$SFA(FT_{sw})$	O(N(ωn+wlog(w)+ωlog(α))

**Table 4:** Summary of dataset characteristics used in experiments. The Jogging and Biking activities (marked with *) were removed from our experiments. The gyroscope and magnetometer sensors were used only in specific scenarios.

Dataset	SHOAIB SH	WISDM	UCI
Individuals	10	19 (36)	30
Hz	50	20	50
Sensors	Accelerometer, Gyroscope *, Magnetometer *	Accelerometer	Accelerometer, Gyroscope *
Location	Belt, Left Pocket, Right Pocket, Upper Arm, Wrist	Belt	Belt
Activities used	Walking, Running, Sitting, Standing, Walking Upstairs, Walking Downstairs, Jogging *, Biking *	Walking, Jogging, Sitting Standing, Walking Upstairs, Walking Downstairs	Walking, Lying Down, Sitting, Standing, Walking Upstairs, Walking Downstairs

**Table 5:** List of all features used in the experiments with the baseline TF.

Domain	Features
Time	min, max, amplitude, amplitude peak, sum, absolute sum, Euclidean norm, mean, absolute mean, mean square, mean absolute deviation, sum square error, variance, standard deviation, Pearson coefficient, zero crossing rate, correlation, cross-correlation, auto-correlation, skewness, kurtosis, area, absolute area, signal magnitude mean, absolute signal magnitude mean, magnitude difference function
Frequency	Energy, energy normalized, power, centroid, entropy, DC component, peak, coefficient sum

**Table 6:** The parameters used in HAR-SR, TF, SAX-VSM, and BOSS-VS algorithms. *TF is a classification method that uses time and frequency domain features.

Algorithm	Features	Datasets	Classification Algorithm	Distance Measure	Parameters
HAR-SR	15	SHOAIB, WISDM, UCI	K-NN (k = 3)	Cosine similarity	Data Fusion = Concatenation Symbolic = SAX ω=6 α=6 T=128 w=50%
*TF	145	SHOAIB, WISDM, UCI	K-NN (k = 3)	Euclidean distance	-
SAX-VSM	46.656	SHOAIB, WISDM, UCI	K-NN (K = 1)	Cosine similarity	Data Fusion = Concatenation Symbolic = SAX ω=6 α=6 T=128 w=50%
BOSS-VS	46.656	SHOAIB, WISDM, UCI	K-NN (K = 1)	Cosine similarity	Data Fusion = Concatenation Symbolic = SAX ω=6 α=6 T=128 w=50%

**Table 7:** Parameter variation of word size and alphabet size.

Dataset	SHOAIB
Data Fusion Methods	Magnitude
Sensors	Accelerometer, Gyroscope, Magnetometer
Symbolic Methods	SAX, SFA
Word Size	4, 6
Alphabet Size	4, 6, 8
Window Size	50% (Slide Window)
Segment Size	2.5 s

**Table 8:** Variation of word size and alphabet size parameters.

Word (ω)	Alphabet (α)	Word Space	Interval
4 (a, b, c, d)	4 (a, b, c, d)	256	aaaa-dddd
4 (a, b, c, d)	8 (a, b, c, d, e, f, g, h)	4096	aaaa-hhhh
6 (a, b, c, d, e, f)	4 (a, b, c, d)	4096	aaaaaa-dddddd
6 (a, b, c, d, e, f)	6 (a, b, c, d, e, f)	46.656	aaaaaa-ffffff
8 (a, b, c, d, e, f, g, h)	4 (a, b, c, d)	65.536	aaaaaaaa-dddddddd
8 (a, b, c, d, e, f, g, h)	8 (a, b, c, d, e, f, g, h)	16777216	aaaaaaaa-hhhhhhhh

**Table 9:** Sensors arrangement for evaluating scenario *A*.

Sensor Group	
SG1	Accelerometer
SG2	Accelerometer, Gyroscope
SG3	Accelerometer, Gyroscope, Magnetometer
SG4	Gyroscope
